# Anti-ceramide single-chain variable fragment mitigates radiation GI syndrome mortality independent of DNA repair

**DOI:** 10.1172/jci.insight.145380

**Published:** 2021-04-22

**Authors:** Jimmy A. Rotolo, Chii Shyang Fong, Sahra Bodo, Prashanth K.B. Nagesh, John Fuller, Thivashnee Sharma, Alessandra Piersigilli, Zhigang Zhang, Zvi Fuks, Vijay K. Singh, Richard Kolesnick

**Affiliations:** 1 Laboratory of Signal Transduction, Sloan Kettering Institute, Memorial Sloan-Kettering Cancer Center, New York, New York, USA.; 2 Laboratory of Comparative Pathology, Rockefeller University, Weill Cornell Medicine and Memorial Sloan-Kettering Cancer Center, New York, New York, USA.; 3 Department of Epidemiology and Biostatistics and; 4 Department of Radiation Oncology, Memorial Sloan-Kettering Cancer Center, New York, New York, USA.; 5 Champalimaud Centre for the Unknown, Lisbon, Portugal.; 6 Division of Radioprotectants, Department of Pharmacology and Molecular Therapeutics, F. Edward Hébert School of Medicine, and; 7 Armed Forces Radiobiology Research Institute, Uniformed Services University of the Health Sciences, Bethesda, Maryland, USA.

**Keywords:** Stem cells, Vascular Biology, Apoptosis, DNA repair, Endothelial cells

## Abstract

After 9/11, threat of nuclear attack on American urban centers prompted government agencies to develop medical radiation countermeasures to mitigate hematopoietic acute radiation syndrome (H-ARS) and higher-dose gastrointestinal acute radiation syndrome (GI-ARS) lethality. While repurposing leukemia drugs that enhance bone marrow repopulation successfully treats H-ARS in preclinical models, no mitigator potentially deliverable under mass casualty conditions preserves GI tract. Here, we report generation of an anti-ceramide 6B5 single-chain variable fragment (scFv) and show that s.c. 6B5 scFv delivery at 24 hours after a 90% lethal GI-ARS dose of 15 Gy mitigated mouse lethality, despite administration after DNA repair was complete. We defined an alternate target to DNA repair, an evolving pattern of ceramide-mediated endothelial apoptosis after radiation, which when disrupted by 6B5 scFv, initiates a durable program of tissue repair, permitting crypt, organ, and mouse survival. We posit that successful preclinical development will render anti-ceramide 6B5 scFv a candidate for inclusion in the Strategic National Stockpile for distribution after a radiation catastrophe.

## Introduction

With the possibility of a radiation disaster by way of nuclear detonation, accident, or radiation dispersal device, urgency for safe and effective medical radiation countermeasures (MRMs) to mitigate radiation toxicities in the general population represents an unmet medical need (1). The Project BioShield Act and Department of Health and Human Services estimate that a MRM must be effective when administered at 24 hours after a nuclear disaster, as this represents the anticipated minimum time necessary to mobilize treatment by first responders to a significant portion of the population of a major city. This timing presents a conceptual challenge, as DNA repair, currently regarded as the exclusive event determining outcome of radiation damage to human tissue (2, 3), would likely be complete at 24 hours after the range of doses leading to the gastrointestinal acute radiation syndrome (GI-ARS; also known as the radiation GI syndrome [RGS]) (4, 5). Hence, if DNA repair determines intestinal stem cell (ISC) survival after radiation exposure, delivery of an effective GI-ARS mitigator 24 hours after irradiation might prove to be impossible. To date, a number of agents, including our anti-ceramide 2A2 Ab (6), effectively protect against GI-ARS lethality in murine models when delivered as prophylaxis. However, being an effective protector does not necessarily predict efficacy as a radiation mitigator (7). In fact, we show here that the clinically approved antioxidant amifostine (7) is a highly effective radioprotector of small intestinal crypt survival in the Withers and Elkind clonogenic assay (8); however, it fails to mitigate crypt lethality. Note, the Withers and Elkind assay directly quantifies radiation dose-dependent lethality of the small intestinal crypt compartment, is predictive of eventual animal death from GI-ARS, and is considered a “gold standard” in the radiation field for evaluating normal tissue response to ionizing radiation.

In this report, we describe the generation of a single-chain variable fragment (scFv) that binds and neutralizes ceramide on the GI endothelial cell surface as the first effective GI-ARS mitigator to our knowledge. When administered 24 hours after 15 Gy, anti-ceramide 6B5 scFv significantly decreased microvascular apoptosis, resulting in increased ISC and crypt survival, and reduced mortality from GI-ARS. We found that 6B5 scFv administration indeed occurred after DNA damage repair (DDR) was complete, and, hence, ISC preservation is independent of DDR. We identify, to our knowledge, a previously unrecognized vascular pathophysiology as a target of anti-ceramide 6B5 scFv mitigation. We propose that successful preclinical development will render anti-ceramide 6B5 scFv a candidate for inclusion in the Strategic National Stockpile for distribution in the event of a nuclear catastrophe.

## Results

**Figure 1:**
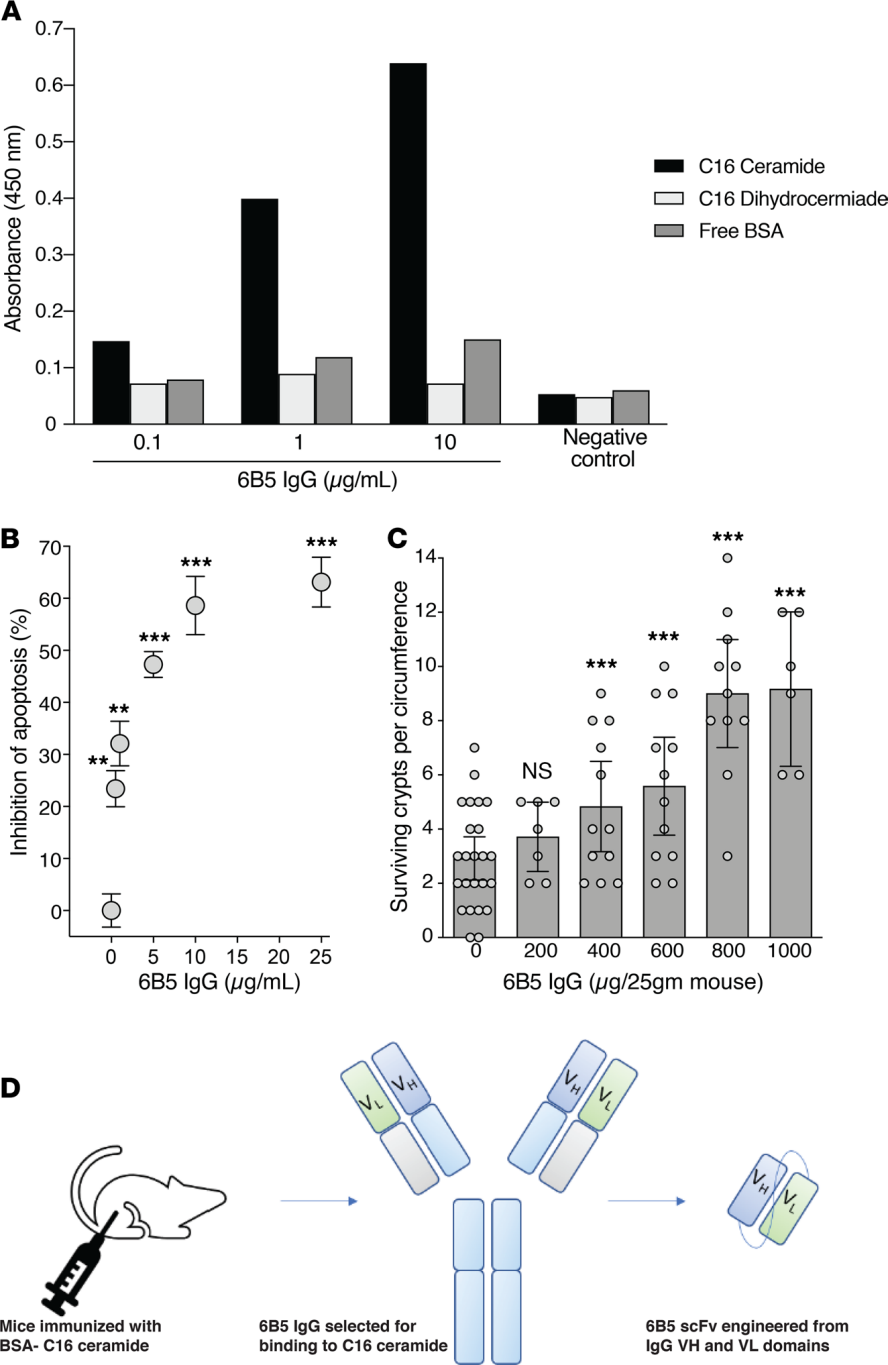
Generation of anti-ceramide 6B5 scFv. (**A**) Monoclonal Ab 6B5 was generated from mice immunized with BSA-conjugated C16-ceramide. Antigen binding was determined using plates coated with 300 ng/ml C16-ceramide, C16-dihydroceramide, or BSA. Purified monoclonal Ab (0.1, 1, and 10 μg/ml) preferentially binds to C16-ceramide. Negative control preimmune serum did not display antigen binding by ELISA. Data show 1 representative experiment of 3 independent experiments. (**B**) Jurkat cells (5 × 10^5^/point) were pretreated with or without the indicated concentrations of 6B5 IgG at 15 minutes before 10 Gy. After fixation and staining with bisBenzimide Hoechst 33258, apoptotic death was quantified by fluorescence microscopy at 16 hours, the optimal time for detecting radiation-induced apoptosis in this cell line (3). Data (mean ± 95% CI) were collated from 3 experiments in which 400 cells were analyzed per point. **P < 0.01, ***P < 0.001 vs. control, unpaired *t* test and multiple-comparison correction using Bonferroni-Dunn correction (α = 0.01). (**C**) Mouse monoclonal Ab 6B5 dose-dependently protects small intestinal crypts. Purified 6B5 IgG (0–1000 μg) was administered to C57BL/6 mice 15 minutes before 15 Gy WBR. Crypt survival was quantified according to the Withers and Elkind microcolony assay (8). Data are from 1 representative experiment of 3 independent experiments with 2 mice each, analyzing 10–20 intestinal circumferences/ mouse. Only continuous, fully intact circumferences were counted. ***P < 0.001 vs. control, Wilcoxon’s rank sum test with continuity correction. (**D**) Schematic shows the generation of 6B5 scFv. Briefly, the VH and VL regions of mouse monoclonal 6B5 Ab were codon optimized and cloned into expression vector pX containing a 6x-His tag and a 12x-glycine linker.

Previous studies demonstrated that neutralization of ceramide is an effective strategy for prevention of GI-ARS (6). Although 2A2 anti-ceramide Ab demonstrated significant potential as an MRM (6), it is effective only by i.v. injection and further was determined to be a poor expressor deemed inappropriate for clinical development. Here, we sought to develop an anti-ceramide scFv MRM effective when dosed by s.c. injection, a route of administration that would enable nonmedical professionals to treat large numbers of people in the event of a radiological disaster. Hence, initial experiments generated a new mouse IgG anti-ceramide monoclonal Ab by immunization of BALB/c mice with a synthetic BSA-conjugated ceramide antigen. Clone 6B5 was selected following screening of Ab-containing hybridoma supernatants for ceramide binding by solid-state ELISA (Figure 1A). To evaluate biologic activity, purified murine anti-ceramide 6B5 IgG was added to culture medium of human Jurkat T lymphocytes 15 minutes before 10 Gy ionizing radiation exposure, an assay standardized in our laboratory for measuring ceramide-mediated apoptosis. 6B5 Ab dose-dependently inhibited radiation-induced ceramide-mediated apoptosis of Jurkat T cells (Figure 1B). Further, i.v. administration of 8–40 mg/kg 6B5 monoclonal Ab to C57BL/6 mice 15 minutes before a 90% lethal dose (LD_90_) of 15 Gy whole-body radiation (WBR) dose-dependently increased crypt survival, as quantified by the microcolony assay of Withers and Elkind at 3.5 days after irradiation (8). Unirradiated C57BL/6 mice display 122 ± 5 crypts/intestinal circumference (5), reduced here after the LD_90_ dose of 15 Gy to 2.9 ± 0.4 crypts/intestinal circumference, consistent with published data (9, 10). Anti-ceramide 6B5 IgG dose-dependently increased surviving crypts after 15 Gy, reaching 9.2 ± 1.1 crypts/intestinal circumference (*P* < 0.001 vs. irradiated alone) at 1 mg/25 g mouse (Figure 1C). Note, the literature indicates that approximately 8%–10% crypt survival in this assay is consistent with small intestinal and mouse survival (5, 11).

**Figure 2:**
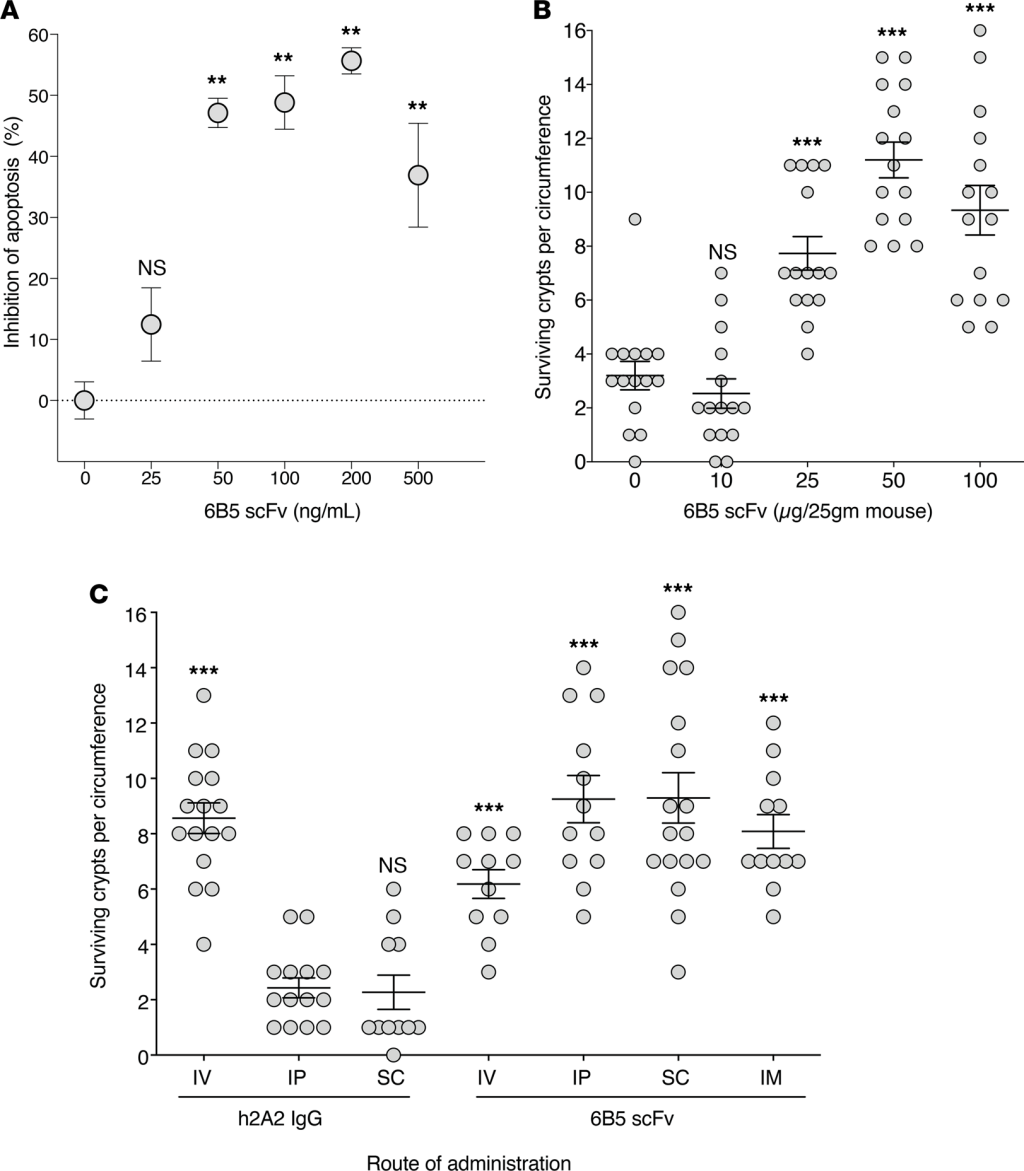
Anti-ceramide 6B5 scFv protects against radiation-induced crypt lethality. (**A**) Anti-ceramide 6B5 scFv inhibits Jurkat cell apoptosis when administered to cell culture medium before 10 Gy exposure, as quantified in Figure 1B. **P < 0.01 vs. control, Wilcoxon’s rank sum test with continuity correction. Data are compiled from 3 independent experiments. (**B**) Anti-ceramide 6B5 scFv inhibits crypt lethality, determined by the microcolony assay, as in Figure 1C, when administered by i.v. infusion to C57BL/6 mice 15 minutes before 15 Gy WBR. ***P < 0.001 vs. control, Wilcoxon’s rank sum test with continuity correction. (**C**) Anti-ceramide 6B5 scFv (100 μg/25 g mouse) delivers equal or greater protection from crypt lethality when administered by the i.p., s.c., or i.m. route compared with i.v. injection. ***P < 0.001 vs. i.p. h2A2 IgG (1 mg/25 g mouse), Wilcoxon’s rank sum test with continuity correction. Data from microcolony assays show 1 representative experiment of 3 experiments, with 2 mice each, analyzing 10–20 intestinal circumferences per mouse. Only continuous, fully intact circumferences were counted.

The Project BioShield Act mandates that MRMs for mass casualty events, such as a radiological disaster, be ready for widespread administration in the field by nonmedical professionals, thereby requiring administration by routes other than i.v. injection (12). As such, full-length Abs would not qualify as effective MRMs for radiation mitigation. To generate an anti-ceramide MRM, an scFv capable of being administered by s.c. injection was engineered from 6B5 by cloning variable regions from the mouse hybridoma (Figure 1D). Heavy chain (VH) and light chain (VL) variable regions of immunoglobulin genes from 6B5 mouse hybridoma cells were sequenced, and a humanized scFv was designed via complementarity-determining region grafting with retention of mouse framework region residues identified by primary sequence analysis. This scFv was cloned with a 6x-His tag in the linker region and expressed in *E*. *coli*. Purified scFv was tested for anti-ceramide biologic activity. Initial experiments confirmed that anti-ceramide 6B5 scFv, similar to parental 6B5 IgG and humanized 2A2 anti-ceramide Ab (6), delivered at 15 minutes before irradiation dose-dependently protected against radiation-induced Jurkat cell apoptosis (Figure 2A) and enhanced crypt survival in C57BL/6 mice, as quantified by the microcolony assay of Withers and Elkind (8) (Figure 2B). Critically, whereas full-length anti-ceramide 2A2 and 6B5 Abs are biologically active only by the i.v. route (Figure 2C and data not shown, respectively), anti-ceramide 6B5 scFv (100 μg/25 g mouse) increased crypt survival via i.p., s.c., or i.m. injection in addition to i.v. infusion (Figure 2C; *P* < 0.001 each vs. h2A2 i.p.).

**Figure 3:**
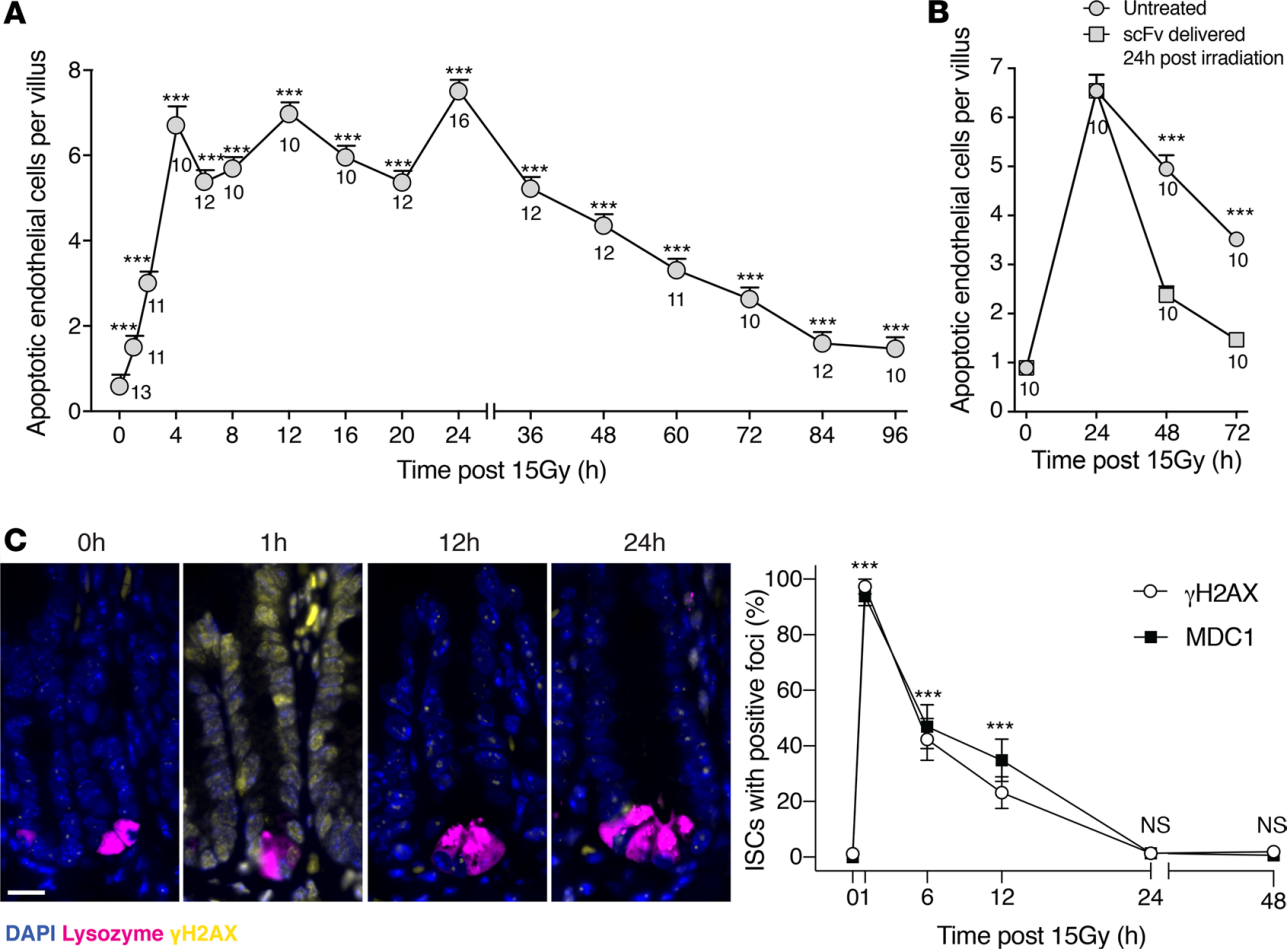
Anti-ceramide 6B5 scFv mitigates ceramide-mediated microvascular endothelial apoptosis after DNA repair is complete. (**A**) Endothelial apoptosis was identified at the indicated times by microscopic detection of TUNEL/MECA-32 double-positive endothelial cells, as previously described (1). Data represent mean ± SEM of apoptotic endothelial cells/villus unit collated from 10–16 mice (numbers below individual data points)/time point, analyzing approximately 20 intact villi/mouse, as in ref. 1. ***P < 0.001 vs. control, Wilcoxon’s rank sum test with continuity correction. (**B**) Administration of 6 mg/kg anti-ceramide 6B5 scFv at 24 hours after 15 Gy WBR mitigates endothelial apoptosis detected at 48 hours and 72 hours after irradiation. ***P < 0.001 vs. untreated, Wilcoxon’s rank sum test with continuity correction. (**C**) Representative immunofluorescence images of γH2AX staining of small intestines from control and irradiated mice at the indicated times after 15 Gy WBR (scale bar: 20 μm) and quantitation of MDC1 and γH2AX foci within crypt intestinal stem cells (ISCs) that reside between Paneth cells at position 1–4 from the crypt base. DAPI staining depicts nuclei and lysozyme staining depicts Paneth cells. Data represent mean ± SEM, with 5 mice/group. ***P < 0.001 vs. control, Wilcoxon’s rank sum test with continuity correction.

Our published data show that microvascular apoptosis peaks at 4–6 hours after 15 Gy in C57B/L6 mice (13) and indicate that 2A2 anti-ceramide Ab pretreatment significantly attenuates this apoptotic response (6). Little is known about the kinetics of endothelial cell death beyond this time frame, including at the 24-hour time point designated by federal guideline as required for mitigation. Figure 3A demonstrates that while apoptosis indeed peaks at 20-fold of background at 4 hours after 15 Gy, the response unexpectedly plateaus at this high level for at least 24 hours, slowly returning thereafter toward baseline by 3.5 days, the time when regenerative small intestinal crypts are quantified using the Withers and Elkind clonogenic assay (8). Administration of anti-ceramide 6B5 scFv at 24 hours after 15 Gy, a time when apoptosis remains at peak levels, resulted in a steep decline in endothelial apoptosis, with 75% reduction by 48 hours and abolition by 72 hours after radiation exposure (Figure 3B). These data indicate that microvascular apoptosis persists at peak levels for 24 hours after 15 Gy WBR and that 6B5 scFv mitigates additional microvascular apoptosis when administered at the 24-hour time point.

Classic radiation biologic concepts consider efficiency of DNA DSB repair as the critical element determining outcome of radiation damage to mammalian tissue, with DNA misrepair or unrepair leading to cell death from evolving prolethal chromosomal aberrations early after radiation cell division (14). Mammalian DNA repair is considered to occur exclusively during the cell cycle arrest phase of the DNA damage response, which ensues immediately after irradiation (2–4). Most investigators believe that irradiated mammalian tissues growth arrest for approximately 1 h/Gy delivered, a fact recently validated in irradiated small intestines (5), before reentering the cell cycle. Thus, our observation that anti-ceramide 6B5 scFv effectively mitigates ongoing endothelial apoptosis when delivered at 24 hours after irradiation, likely after DNA repair is complete, poses a scientific conundrum and defies expectations. To better understand how the kinetics of DSB repair after the LD_90_ dose of 15 Gy relate to mitigation of microvascular apoptosis, we examined the accrual and resolution of γH2AX and MDC1 repair foci, together considered as surrogate for number and repair of DSBs, respectively (15). As anticipated, accrual of MDC1 and γH2AX foci within the crypt base columnar (CBC) small ISC compartment, considered vital for crypt survival (5, 16), peaks within 1 hour of 15 Gy exposure and resolves completely by 24 hours (Figure 3C and Supplemental Figure 1; supplemental material available online with this article; https://doi.org/10.1172/jci.insight.145380DS1). Hence, anti-ceramide 6B5 scFv delivered at 24 hours after 15 Gy, when DNA repair is complete, could not mitigate GI-ARS lethality by enhancing DNA repair. Note that our recently published analysis of the pattern of growth arrest and reinitiation after 15 Gy by EDU staining shows that cell cycling reinitiates at 12–18 hours in small intestinal CBC cells of C57BL/6 mice (16).

**Figure 4:**
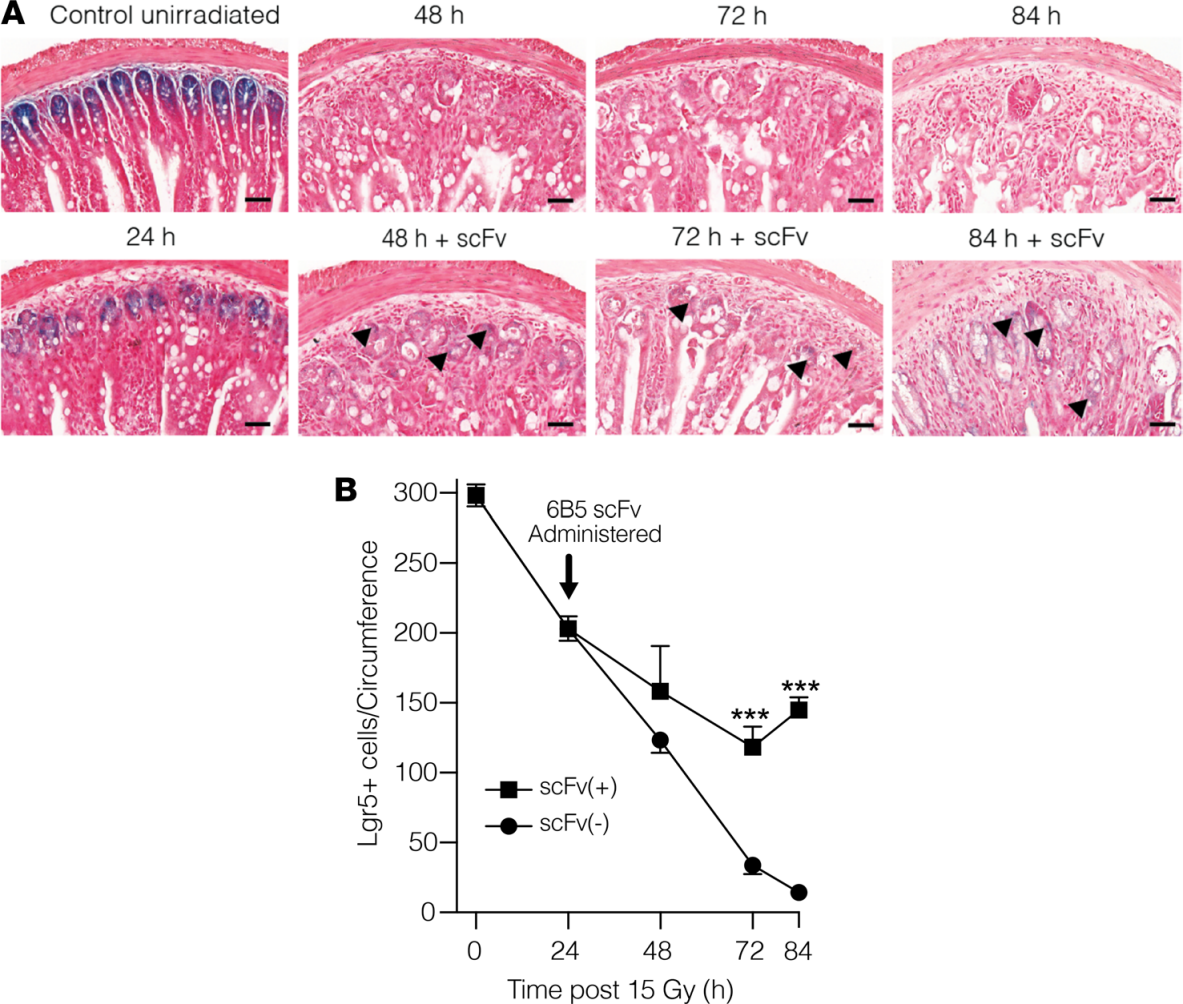
Anti-ceramide 6B5 scFv mitigates loss of Lgr5^+^ intestinal stem cells in Lgr5-LacZ reporter mice. (**A**) Impact of 6B5 scFv on the time course of radiation-induced loss of Lgr5^+^-LacZ intestinal stem cells (ISCs). Representative LacZ staining of 5 μM proximal jejunum sections from 15 Gy–treated mice with or without s.c. 6B5 scFv (150 μg/25 g mouse) administered 24 hours after radiation. Black arrowheads delineate Lgr5^+^ (blue) cells within crypts. Note that radiation injury reduces blue signal intensity compared with unirradiated controls, which does not fully recover for at least 10 days after irradiation (2). Scale bars: 40 μm. (**B**) Quantitation of Lgr5^+^ cells in irradiated mice. Data (mean ± SD) were collated from 7 mice/group, evaluating 5 circumferences per mouse. ***P < 0.001 vs. mice irradiated with 15 Gy WBR without scFv, with Bonferroni’s correction (threshold: α = 0.05/5 = 0.01).

**Figure 5:**
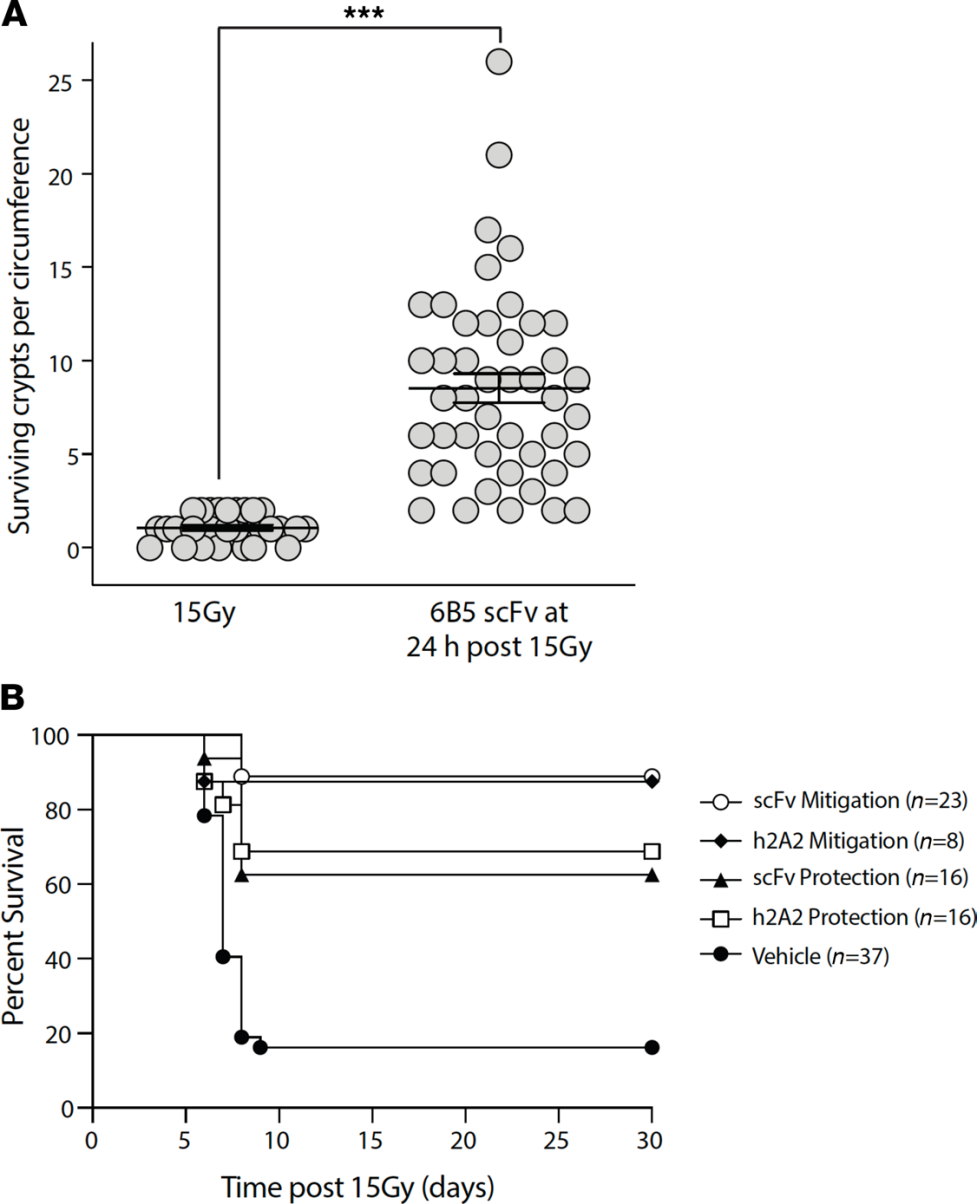
Anti-ceramide 6B5 scFv mitigates radiation GI syndrome mortality. (**A**) Delivery (i.v.) of anti-ceramide 6B5 scFv (5 mg/kg) at 24 hours after 15 Gy WBR mitigates small intestinal crypt lethality in C57BL/6 mice measured by the Withers and Elkind assay at 3.5 days after radiation (8). ***P < 0.001, Wilcoxon’s rank sum test with continuity correction. Data are compiled from 3 independent experiments. (**B**) Anti-ceramide 6B5 scFv (5 mg/kg) delivered (s.c.) at 15 minutes before irradiation (Protection) or at 24 hours after irradiation (Mitigation) prevents the lethal GI-ARS, as determined by increased 30-day survival of C57BL/6 mice exposed to 15 Gy WBR and administered HSCT (3 × 10^6^ cells), as calculated by the product limit Kaplan-Meier method. Numbers in parentheses represent animals/group. *P* < 0.001 h2A2 (40 mg/kg) or scFv mitigation vs. vehicle, *P* < 0.01 h2A2 or scFv protection vs. vehicle, 2-sided Fisher’s exact test.

Investigations using Lgr5-LacZ reporter mice, which mark Lgr5^+^ ISCs, indicate that the impact of mitigation of microvascular apoptosis occurs at the level of the ISC. Despite delivery after DNA repair is complete, 6B5 scFv injection at 24 hours after 15 Gy results in mitigation of Lgr5^+^ ISC lethality (Figure 4A). Analysis of proximal jejunal sections indicated that ISCs decrease from a baseline of 295 ± 9/circumference in unirradiated animals to 206 ± 11/circumference at 24 hours after 15 Gy and then progressively to 15 ± 3/circumference at 84 hours after irradiation, the time at which the Withers and Elkind crypt survival analysis is routinely performed (8). Administration of 6B5 scFv at 24 hours after 15 Gy effectively mitigated this loss of Lgr5^+^ ISCs (Figure 4B; *P* < 0.001 vs. 72 hours and 84 hours after 15 Gy). Consistent with this observation, 6B5 scFv injection at 24 hours after 15 Gy yields dose-dependent mitigation of crypt lethality measured at 3.5 days, resulting in crypt survival similar to what was observed following prophylactic administration of parental 6B5 IgG (Figure 2, A and B). The number of surviving crypts/intestinal circumference increased from 1.1 ± 0.1 in 15 Gy–irradiated mice to 8.5 ± 0.8 in 6B5 scFv-treated animals at 24 hours following 15 Gy WBR (Figure 5A). While it might be inferred that because 6B5 scFv effectively protects against crypt lethality when administered before 15 Gy WBR, it would similarly mitigate crypt lethality when dosed 24 hours after radiation exposure, the cytoprotective adjuvant amifostine proves that protection against GI-ARS did not predict mitigation, as amifostine, while effective when delivered as prophylaxis, was ineffective in mitigating crypt lethality when administered at 24 hours after 15 Gy WBR (Supplemental Figure 2).

Mitigation of intestinal microvascular endothelial apoptosis, Lgr5^+^ ISC depletion, and crypt lethality saved C57BL/6 mice from lethal consequences of the GI-ARS (Figure 5B). Kaplan-Meier survival was monitored in mice exposed to 15 Gy WBR and administered 3 × 10^6^ syngeneic hematopoietic stem cells to prevent bone marrow aplasia. Whereas only 16% of animals survived beyond the first 8 days of 15 Gy exposure, despite bone marrow transplant, mice receiving s.c. anti-ceramide scFv either 15 minutes before or at 24 hours following 15 Gy exposure demonstrated 61% and 87% 30-day survival, respectively. Full-length (i.v.) anti-ceramide 2A2 IgG yielded virtually identical outcomes. In a subset of anti-ceramide–treated mice studied for longer times, more than 90% of those surviving 30 days were alive at 90 days without evidence of significant tissue damage at autopsy (Supplemental Table 1). These data indicate that 6B5 scFv confers mitigation of GI-ARS lethality that is both durable and safe.

## Discussion

The current study identifies anti-ceramide 6B5 scFv as possibly the first effective mitigator of the lethal GI-ARS capable of being delivered under mass casualty conditions. Administration of 6B5 scFv (s.c.) at 24 hours following a lethal GI-ARS dose of ionizing radiation attenuates microvascular endothelial cell apoptosis within the crypt villus unit, thereby promoting ISC survival and protecting from crypt lethality and organ damage beyond the capacity for regeneration. While previous studies demonstrate effective protection from GI-ARS with humanized full-length anti-ceramide 2A2 Ab, we show here that mitigation cannot be assumed for radioprotective agents, as amifostine, which is highly effective when delivered as prophylaxis, does not offer any benefit when administered 24 hours after 15 Gy WBR. The extraordinary success observed in mitigating ongoing endothelial cell death is unanticipated, as anti-ceramide 6B5 scFv has a plasma half-life in hours (*t*_1/2α_ = 2.3 hours and *t*_1/2β_ = 10.8 hours; Scheinberg and Kolesnick, unpublished observations), and hence, its efficacy extends well beyond the time of its clearance from the systemic circulation. We posit that during evolution of parenchymal tissue damage after irradiation, ASMase/ceramide-dependent endothelial apoptosis likely represents a feed-forward process, which, once disrupted, even for a brief period, initiates a previously unknown tissue reparative process that is both robust and durable. This prolonged protection following treatment is reminiscent of the impact of Lucentis, a fab fragment of bevacizumab, which, despite a short half-life (in days), provides a durable therapeutic outcome (for months), mitigating microvascular damage and preventing further pathology in diabetic macular edema after only a few injections (17).

Another surprising aspect of anti-ceramide 6B5 scFv mitigation of the GI-ARS is that at the time of administration at 24 hours after irradiation, small intestine crypt epithelial cells (including Lgr5^+^ CBC stem cells) have already growth arrested, completed DSB repair, and returned to cell cycling (16). While in vivo evidence, corroborated by organoid data, indicate that CBC stem cell survival is determinant in organ (small intestinal) survival (5, 16, 18), detailed analysis of loss of CBC cells and their regeneration reveals that approximately one-third die by apoptosis during the growth arrest DNA reparative phase during day 1 after potentially lethal irradiation, while two-thirds die during the rapid regenerative phase occurring at 24–48 hours after irradiation (16, 19). Indeed, we observed similar results in this study. These effects precede crypt loss, which occurs at 48–72 hours after irradiation and crypt regeneration, which peaks at 84 hours after irradiation (5, 19). Whether anti-ceramide 6B5 scFv mitigation of endothelial cell death delivered at 24 hours after irradiation protects ISCs from mitotic death, secondary injury from ongoing tissue damage, or enhances their regeneration is a topic of ongoing investigation in our laboratory.

In sum, our data define anti-ceramide 6B5 scFv as the first, to our knowledge, highly effective mitigator of GI damage following the high-dose radiation exposure that might be incurred during a nuclear catastrophe. Based upon the current data set, we posit that 6B5 scFv fulfills the mandate of the US Department of Health and Human Services Public Health Emergency Medical Countermeasures Enterprise (20), a government health security team tasked to address the medical response for American civilian and military casualties in case of the deliberate use of weapons of mass destruction, consistent with the goals of the President’s Biodefense for the 21st Century and National Strategy for Medical Countermeasures against Weapons of Mass Destruction directives (21). Assuming successful development of 6B5 scFv as mitigator of the GI-ARS in rhesus macaques in accordance with the “animal rule” protocol (1), 6B5 scFv might be a viable candidate for inclusion as a MRM in the Strategic National Stockpile, a repository that manages medical resources for distribution to disaster-affected areas (22).

## Methods

Further information can be found in Supplemental Methods.

### Ab generation and production.

BALB/c mice were immunized by multiple i.p. administrations of BSA-conjugated C_16_-ceramide (days 1, 14, 28, 56, 84, and 112). Titer was monitored by ELISA to assess immune response. Harvested splenocytes were fused with mouse myeloma cells (P3X63Ag8.653, ATCC) at a 4:1 ratio using polyethylene glycol (MW 1500). After fusion, cells were seeded and cultured in 96-well plates at 1 × 10^5^ cells/well in RPMI 1640 selection medium containing 20% fetal bovine serum, 10% Hybridoma supplements (MilliporeSigma), 2 mM L-glutamine, 100 U/ml penicillin, 100 μg/ml streptomycin, 10 mM HEPES, and 1× hypoxanthine-aminopterin-thymidine (MilliporeSigma). Hybridoma supernatants were screened by ELISA using BSA-conjugated C_16_-ceramide. Selected hybridoma were subcloned 4 times by limited dilution and screened by ELISA, as previously published (6). 6B5 IgG was purified from ascites using immobilized mannan-binding protein beads.

### scFv generation and production.

Total RNA was extracted from frozen hybridoma cells and RNA was reverse transcribed into cDNA using isotype-specific anti-sense primers or universal primers (GenScript) following the SuperScript III First-Strand Synthesis System technical manual. The Ab fragments of VH, VL, CH, and CL were amplified according to the standard operating procedure for RACE at GenScript. Amplified Ab fragments were separately cloned into a standard cloning vector using standard molecular cloning procedures. Clones with inserts of correct sizes were identified by PCR screening. No less than 10 single colonies with correct size inserts were sequenced for each Ab fragment. Codon optimization and gene synthesis of 6B5 scFv was performed. The resulting scFv fragment was cloned directionally into the pX expression vector. The DNA sequence of the construction was confirmed before expression. A single colony was inoculated in 200 ml 2×YT medium supplemented with 200 μg/ml Ampicillin (GenScript) and subsequently incubated at 37°C with 220 rpm shaking overnight. About 200 ml of the overnight culture was expanded in 20 L of 2×YT medium supplemented with 200 μg/ml Ampicillin and incubated at 37°C with 220 rpm shaking. Protein expression was induced by the addition of 1 mM IPTG at an optical density of 0.5–0.6 and supplemented with 0.2% glycerol. The induced culture was grown for 20 hours at 27°C with 220 rpm shaking. 6B5 scFv was purified using Ni-NTA affinity chromatography and standard protocols.

### Statistics.

Wilcoxon’s rank sum test with continuity correction or Fisher’s exact test was performed with R software. Unpaired 2-tailed *t* test multiple-comparison correction using Bonferroni-Dunn correction was performed with GraphPad Prism version 8.4.3. *P* values of less than 0.05 were considered significant, while *P* > 0.05 was considered not significant.

### Study approval.

Animal experimental protocols were approved by the Memorial Sloan-Kettering Cancer Center Research Animal Resource Center.

## Author contributions

JAR and VKS performed the 6B5 scFv studies. SB performed DNA repair experiments. CSF performed apoptosis experiments. JF and TS performed survival experiments. PKBN performed stem cell studies. AP is a board-certified animal pathologist who performed the autopsies. ZZ is a biostatistician who analyzed the data. JAR and RK wrote the manuscript. JAR, VKS, ZF, and RK provided overall scientific direction.

## Supplementary Material

Supplemental data

## Version 1 03/16/2021

In-Press Preview

## Version 2 04/22/2021

Electronic publication
