# Supplementary material for: Anti-ceramide single-chain variable fragment mitigates radiation GI syndrome mortality independent of DNA repair
Source: JCI Insight. 2021 Apr 22;6(8):e145380. doi: 10.1172/jci.insight.145380 (PMC8119204; doi:10.1172/jci.insight.145380)
Supplement: Supplemental data [file jciinsight-6-145380-s157.pdf]

Supplementary Materials for

Anti-Ceramide Single-chain Variable Fragmnet Mitigates Radiation GI Syndrome Mortality Independent of DNA Repair

Jimmy A. Rotolo, Chii Shyang Fong, Sahra Bodo, Prashanth Kumar, John Fuller, Thivashnee Sharma, Alessandra Piersigilli, Zhigang Zhang, Zvi Fuks, Vijay K. Singh, Richard Kolesnick*

*Correspondence: E-mail: r-kolesnick@ski.mskcc.org

This PDF file includes:

Methods
Fig. S1
Fig. S2
Table S1

METHODS

Radiation source: Radiation was delivered to C57BL/6J mice (The Jackson Lab #000664) using a Shepherd Mark-I unit (Model 68, SN643) operating a ^{137}Cs source at a dose rate of 2.12 Gy/min. For experiments involving events occurring under 10 min radiation was delivered at 13.1 Gy/min. For protection studies, pre-treatment with anti-ceramide 6B5 scFv or 2A2 Ab was at 15 min prior to irradiation whereas for mitigation studies treatment was at 24h post irradiation.

Abs used for immunofluorescence DNA repair focus studies: Primary Abs used include: mouse monoclonal Ab against γH2AX-Ser139 (Millipore [clone JBW 301], #05-636, dilution 1:1000), mouse monoclonal anti-MDC1 (Millipore #05-1572 [clone P2B11], dilution 1:100), rabbit polyclonal anti-lysozyme Ab (Novus Biologicals #nbp2-61118, dilution 1:1,000). Secondary Abs: F(ab')2-goat anti-rabbit or anti-mouse IgG (H+L) cross-adsorbed secondary Ab, Alexa Fluor 488 (ThermoFisher #A-11070 #A-11017) 2 mg/ml were used at a dilution of 1:400.

Cell culture: Jurkat T lymphocytes (clone E6-1) were obtained from the ATCC (Rockville, MD). Cells were grown in a 5% CO2 incubator at 37 °C in RPMI 1640 medium supplemented with 10% heat-inactivated fetal bovine serum and 10 mM HEPES (pH 7.4), 2 mM L-glutamine, 1 mM sodium pyruvate, 100 µM nonessential amino acids, 100 units/ml penicillin, and 100 µg/ml streptomycin. Apoptosis was measured by morphologic examination following Hoechst 33258 *bis*-benzimide staining as published (1).

Survival of mice after whole body irradiation: Actuarial survival of animals was calculated by the product limit Kaplan-Meier method (2). Terminally sick animals

displaying an agonal breathing pattern were sacrificed by hypercapnia asphyxiation and evaluated by necropsy to determine cause of death. Intestinal specimens were fixed in formaldehyde, and stained with hematoxylin, as described (3). GI damage was diagnosed as cause of death when small intestines displayed denuded mucosa with nearly no villae or crypts apparent or when the mucosa displayed limited mucosal repair (3). Note that for every Kaplan Meier study the batch of commercial C57B/L6 mice (The Jackson Laboratory C#000664) used was first subjected to a control dose survival study to confirm the LD_{90} dose as 15Gy. In one of six Kaplan Meier studies reported here however the batch of commercial C57B/L6 mice displayed a slightly lower LD_{90} dose of 14.5Gy. For simplicity, data from this study are collated in Fig. 4B with the remaining studies and referred to generically as 15Gy.

Small intestinal endothelial apoptosis: Apoptosis in the small intestinal lamina propria was determined by double staining with TUNEL for apoptosis and immunostaining using a rat Ab against the endothelial cell surface marker MECA-32 (DSHB # MECA-32-s), as described (4).

DSB repair immunofluorescence focus studies: Investigations examining DNA repair using focus technology were performed as published by us (5). Briefly, paraffin-embedded tissue sections (3μm) were melted on a heat block, de-paraffinized by 3x10 min in xylene, 2x3 min in 100% ethanol, 2x3 min in 95% ethanol, 2x3 min in 70% ethanol, then washed with distilled water and transferred to 1X Phosphate Buffered Saline (PBS). Antigen retrieval was performed in boiled 0.1 M citric acid buffer (pH 6.0) in a Decloaking Chamber (Biocare Medical) at

125˚C for 5 min, cooled down for 20 min at room temperature, washed with distilled water, and transferred to washing buffer containing 0.1% Triton X-100 in 1X PBS for 20 min at room temperature. Blocking with 2% bovine serum albumin acetylated (Sigma Aldrich #B2518) and 10% normal goat serum (ThermoFisher Scientific #PCN5000) in 0.1% Triton X-100 in 1X PBS was for 1h at room temperature. DNA repair foci were probed using murine primary Abs against γH2AX-Ser139 (Millipore [clone JBW 301], #05-636, dilution 1:1000) and MDC1 (Millipore #05-1572 [clone P2B11], dilution 1:100) overnight at 4˚C, followed by anti-mouse IgG (H+L) cross-adsorbed secondary Ab Alexa Fluor 488 (ThermoFisher #A-11070 #A-11017, dilution 1:400). Prolong gold and slow fade anti-fade reagent with DAPI (ThermoFisher Scientific #P36962) were used to protect from photobleaching and quenching of fluorescent signal, respectively.

Microscopy of DSB repair foci: Multi-channel fluorescence images were acquired using an upright wide-field Zeiss Axio2 Imaging Microscope with AxioCam MRm Camera (1360x1036 pixels image array) and 40X2 objective of Zeiss Plan-Neofluar 1.3NA oil dic (1083-997). Exposure time was set based on images of intermediate intensity, avoiding over-saturation from brightest foci. Once exposure time was set, it was kept constant within each set of experiments. Microscopy yields blue DAPI staining of nuclear areas, fluorescent focus staining, and overlay of co-localized images. 5 images containing 3-11 crypts/image were randomly selected from each tissue section for quantitation of focus numbers.

Crypt Microcolony Survival Assay: The Microcolony Survival Assay was performed as described by Withers and Elkind (6, 7).

β-galactosidase (lacZ) staining. 8-10 week old *Lgr5-lacZ* male reporter mice were euthanized after radiation and 2.5 cm segments of proximal jejunum obtained, as published by us (8). Specimens were fixed, stained for the presence of β-galactosidase (lacZ), blocked, sectioned, and counterstained with nuclear fast red as published (8, 9). Numbers of blue Lgr5+ intestinal stem cells were quantified and graphed using ImageJ and Prism9 software, respectively.

REFERENCES

1. Haimovitz-Friedman A, Kan CC, Ehleiter D, Persaud RS, McLoughlin M, Fuks Z, et al. Ionizing radiation acts on cellular membranes to generate ceramide and initiate apoptosis. *J Exp Med.* 1994;180(2):525-35.
2. Kaplan EL, and Meier P. Nonparametic estimation from incomplete observations. *J of the American Statistical Association.* 1958;53:457-48.
3. Rotolo JA, Maj JG, Feldman R, Ren D, Haimovitz-Friedman A, Cordon-Cardo C, et al. Bax and Bak do not exhibit functional redundancy in mediating radiation-induced endothelial apoptosis in the intestinal mucosa. *International Journal of Radiation Oncology, Biology, Physics.* 2008;70(3):804-15.
4. Paris F, Fuks Z, Kang A, Capodieci P, Juan G, Ehleiter D, et al. Endothelial apoptosis as the primary lesion initiating intestinal radiation damage in mice. *Science.* 2001;293(5528):293-7.
5. Bodo S, Campagne C, Thin TH, Higginson DS, Vargas HA, Hua G, et al. Single-dose radiotherapy disables tumor cell homologous recombination via ischemia/reperfusion injury. *J Clin Invest.* 2019;129(2):786-801.
6. Maj JG, Paris F, Haimovitz-Friedman A, Venkatraman E, Kolesnick R, and Fuks Z. Microvascular Function Regulates Intestinal Crypt Response to Radiation. *Cancer Res.* 2003;63(15):4338-41.
7. Withers HR, and Elkind MM. Microcolony survival assay for cells of mouse intestinal mucosa exposed to radiation. *Int J Radiat Biol Relat Stud Phys Chem Med.* 1970;17:261-7.
8. Hua G, Thin TH, Feldman R, Haimovitz-Friedman A, Clevers H, Fuks Z, et al. Crypt base columnar stem cells in small intestines of mice are radioresistant. *Gastroenterology.* 2012;143(5):1266-76.

9. Barker N, van Es JH, Kuipers J, Kujala P, van den Born M, Cozijnsen M, et al. Identification of stem cells in small intestine and colon by marker gene Lgr5. *Nature.* 2007;449(7165):1003-7.

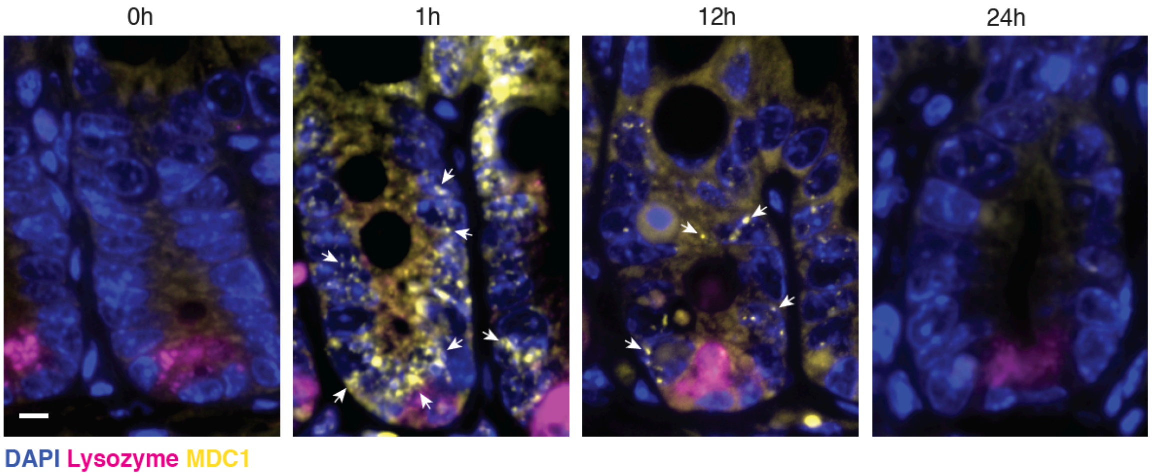

Fig. S1 Representative immunofluorescence images of MDC1 staining of small intestines from control and irradiated mice at the indicated times post 15Gy WBR. Scale 20 μm. Several examples of MDC1 foci are indicated by arrows. Note that when 6B5 anti-ceramide scFv is delivered at 24h post radiation MDC1 foci are fully resolved.

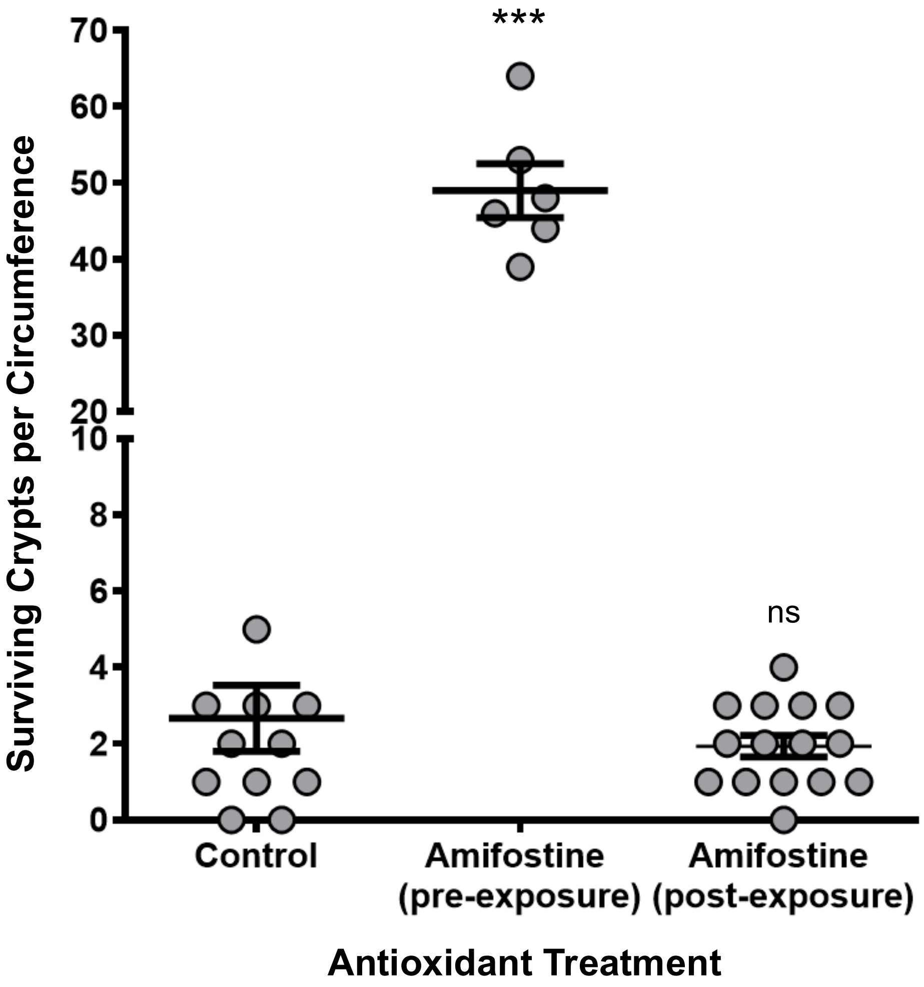

Fig. S2 Quantification of crypt survival following administration of the antioxidant amifostine (WR2721). Amifostine (400 mg/kg by intraperitoneal injection) was administered 15 min prior to or 24 hours following 15Gy whole body radiation. Crypt survival was assessed at 3.5 days post irradiation by the Microcolony Assay of Withers and Elkind. *** $P<0.001$ versus control, unpaired t test.

	RBC & Platelet Indices								
	Control 1	Control 2	scFV 1	scFV 2	h2A2 1	h2A2 2	h2A2 3	h2A2 4	Reference Range
**RBC (M/uL)**	10.13	11.52	9.55	9.86	10.61	10.01	11.76	10	7.14-12.20
**HGB (g/dL)**	14	15.6	13.5	14	14.7	13.9	16.5	14.2	10.8-19.2
**HCT (%)**	50.5	55.7	47.2	49.4	53.3	47.9	57.3	48.4	37.2-62.0
**MCV (fL)**	49.9	48.4	49.4	50.1	50.2	47.9	48.7	48.4	42.6-56.0
**MCH (pg)**	13.8	13.5	14.1	14.2	13.9	13.9	14	14.2	11.7-16.8
**MCHC (g/dL)**	27.7	28	28.6	28.3	27.6	29	28.8	29.3	24.6-35.9
**RDW-SD (fL)**	31.6	30.4	30.8	30.9	31.3	31	28.7	26.9	--
**RDW-CV (%)**	24	25.5	23.3	23.6	24.1	24.6	24.8	22.4	15.9-21.1
**RET# (K/uL)**	819.5	370.9	580.6	432.9	421.2	409.4	306.9	318	294-444
**RET (%)**	8.09	3.22	6.08	4.39	3.97	4.09	2.61	3.18	2.56-4.56
**PLT (K/uL)**	906	1080	826	950	844	701	912	897	565-2159
**PDW (fL)**	7.4	7.8	7.3	7.3	7.2	7.6	7.7	7	--
**MPV (fL)**	6.2	6.2	6.2	6.2	6.1	6.5	6.3	6	4.3-6.1
**Morphology**	3+ polychromasia.	2+ polychromasia. 2+ PLT clumps.	3+ polychromasia.	3+ polychromasia.	2+ polychromasia.	3+ polychromasia.	2+ polychromasia.	2+ polychromasia.	

	Differential								
	Control 1	Control 2	scFV 1	scFV 2	h2A2 1	h2A2 2	h2A2 3	h2A2 4	Reference Range
**Neut# (K/uL)**	1.07	6.91	4.23	4.23	2.64	4.08	2.84	2.15	0.42-3.09
**Band# (K/uL)**	0	0.36	0	0	0	0	0	0	---
**LYMPH# (K/uL)**	8.54	8.73	9.78	12.87	11.89	5.12	4.55	3.82	2.88-11.15
**MONO# (K/uL)**	0.53	0.91	0.29	0	0.99	0	0.32	0.27	0.15-0.94
**EO# (K/uL)**	0.25	0.35	0.04	0	0.36	0	0.2	0.28	0.01-0.50
**BASO# (K/uL)**	0	0	0	0	0	0	0	0	0.00-0.14
**NEUT (%)**	10	38	29	24	16	43	35	32	7.36-28.59
**BAND (%)**	---	2	---	---	---	---	---	---	---
**LYMPH (%)**	80	48	67	73	72	54	56	57	61.26-87.12
**MONO (%)**	5	5	2	---	6	---	4	4	2.18-11.02
**EO (%)**	5	7	2	3	6	3	5	7	0.13-4.51
**BASO%**	---	---	---	---	---	---	---	---	0.01-1.26

	Anatomic Pathology							
	Control 1	Control 2	scFV 1	scFV 2	h2A2 1	h2A2 2	h2A2 3	h2A2 4
	Gross Findings							
	BCS* 3/5. Multifocal brown/grey discoloration of fur coat	BCS 2/5. Multifocal brown/grey discoloration of fur coat	BCS 3/5. Multifocal white discoloration of fur coat	BCS 3/5. Multifocal white discoloration of fur coat	BCS 3/5. Multifocal brown/grey discoloration of fur coat. Multifocal lung hemorrhages	BCS 2/5. Multifocal brown/grey discoloration of fur coat. Enhanced lobular pattern of the liver	BCS 3/5	BCS 2/5. Multifocal brown/grey discoloration of fur coat
	Histopathologic findings							
**Liver**	multifocal minimal perivascular and randomly scattered lymphocytic infiltrates	multifocal minimal to mild perivascular and randomly scattered lymphocytic infiltrates	multifocal minimal to mild perivascular and randomly scattered lymphocytic infiltrates	multifocal minimal perivascular and randomly scattered lymphocytic infiltrates with occasional hepatocellular degeneration	multifocal minimal to mild perivascular and randomly scattered lymphocytic infiltrates with occasional hepatocellular degeneration	multifocal minimal to mild perivascular and randomly scattered lymphocytic infiltrates	multifocal minimal to mild perivascular and randomly scattered lymphocytic infiltrates	multifocal mild perivascular and randomly scattered lymphocytic infiltrates
**Epididymis**	diffuse azoospermia	diffuse oligo-azoospermia	diffuse oligo-azoospermia	diffuse oligo-azoospermia	diffuse oligo-azoospermia	diffuse oligo-azoospermia	diffuse oligo-azoospermia	diffuse oligo-azoospermia
**Testes**	bilateral, multifocal, moderate to severe tubular degeneration with oligozoospermia	bilateral, multifocal, moderate to severe tubular degeneration with oligozoospermia	bilateral, multifocal, moderate to severe tubular degeneration with oligozoospermia	bilateral, multifocal, moderate to severe tubular degeneration with oligozoospermia	bilateral, multifocal, moderate to severe tubular degeneration with oligozoospermia	bilateral, multifocal, moderate to severe tubular degeneration with oligozoospermia	bilateral, multifocal, moderate to severe tubular degeneration with oligozoospermia	bilateral, multifocal, moderate to severe tubular degeneration with oligozoospermia
**Tongue**	multifocal minimal interface glossitis	-	multifocal minimal interface glossitis	multifocal mild interface glossitis	-	-	-	-
**Incisors**	bilateral tooth degeneration	bilateral tooth degeneration	bilateral tooth degeneration	bilateral tooth degeneration	bilateral tooth degeneration	unilateral tooth degeneration	-	unilateral tooth degeneration
**Kidneys**	bilateral, multifocal, minimal cortical tubules regeneration with epithelial dysplasia	bilateral, multifocal, minimal cortical tubules regeneration with epithelial dysplasia	bilateral, multifocal, minimal cortical tubules regeneration with epithelial dysplasia. Multifocal, minimal interstitial lymphocytic infiltration	bilateral, multifocal, minimal cortical tubules regeneration with epithelial dysplasia. Rare hyaline casts	bilateral, multifocal, minimal cortical tubules regeneration with epithelial dysplasia. Rare hyaline casts	bilateral, multifocal, minimal cortical tubules regeneration with epithelial dysplasia. Rare hyaline casts	-	-
**Lungs**	-	-	multifocal, mild perivascular infiltration of lymphocytes	-	-	bilateral, focal alveolar histiocytosis with interstitial mild fibrosis	-	bilateral, focal, alveolar histiocytosis with fibrosis, mild
**Pancreas**	multifocal, minimal interstitial fibrosis, lymphocytic pancreatitis, steatitis and acinar atrophy	multifocal, minimal interstitial fibrosis and lymphocytic pancreatitis and acinar atrophy	multifocal, minimal interstitial fibrosis and lymphocytic pancreatitis, steatitis and and acinar atrophy	multifocal interstitial fibrosis, minimal, and acinar atrophy	diffuse interstitial fibrosis with acinar loss, mild to moderate and lymphocytic steatitis	multifocal, minimal interstitial fibrosis, lymphocytic pancreatitis and acinar atrophy	multifocal interstitial fibrosis with acinar atrophy, minimal	diffuse interstitial fibrosis with acinar loss, moderate with lymphocytic pancreatitis and peripancreatic steatitis and ductal hyperplasia
**Stomach (glandular)**	diffuse lymphocytic gastritis, minimal	multifocal lymphocytic gastritis, minimal	multifocal lymphocytic gastritis, mild. Focal mucosal dysplasia	multifocal lymphocytic gastritis, minimal	multifocal lymphocytic gastritis, minimal	multifocal lymphocytic gastritis, minimal	multifocal lymphocytic gastritis, minimal	-

Supplementary Table S1. Complete Blood Count (CBC with automated differential) and list of gross and microscopic necropsy findings. The hematologic values were within the reference ranges in the majority of the animals. Elevation of the reticulocytic counts in mouse 1 from group scFv is considered incidental as it was present also in the control group. A mild relative neutrophilia and lymphocytopenia were present in 75% (3/4) of mice in group h2A2 suggesting a treatment related effect. All animals had similar macroscopic and histopathologic findings regardless of the anti-ceramide treatment, if administered. The histopathologic changes were in average of minimal to mild severity and of limited to no clinical relevance, except for the oligo-, azoospermia and moderate chronic pancreatitis. Degeneration of the testicular germ cells was induced by irradiation and it is an irreversible impairment of fertility (PMID: 9092926). Fur coat depigmentation and incisors degeneration are also typical changes observed after total body irradiation of mice (https://doi.org/10.1080/09553008114550501; https://doi.org/10.1111/j.1365-2184.1968.tb00321.x). Chronic pancreatitis was more severe in 50% of the mice (2/4) that received h2A2 treatment as it was accompanied by replacement of the acinar units by parenchyma and ductal hyperplasia. The BCS in 1/2 of the affected mice was below the normal reference (2/5). Alveolar histiocytosis is a common background change especially in B6 mice (DOI: 10.1177/0192623309353423) . The incidence in 50% of the h2A2 group is however suggestive of a possible treatment related effect, with no adverse connotation given the focal extension and the very low severity. *BCS= body condition score (PMID: 10403450).
